# Genome-Wide Screening for Genetic Alterations in Esophageal Cancer by aCGH Identifies 11q13 Amplification Oncogenes Associated with Nodal Metastasis

**DOI:** 10.1371/journal.pone.0039797

**Published:** 2012-06-25

**Authors:** Jianming Ying, Ling Shan, Jisheng Li, Lan Zhong, Liyan Xue, Hong Zhao, Lili Li, Cordelia Langford, Lei Guo, Tian Qiu, Ning Lu, Qian Tao

**Affiliations:** 1 Department of Pathology, Cancer Hospital, Peking Union Medical College & Chinese Academy of Medical Sciences, Beijing, China; 2 Cancer Epigenetics Laboratory, Department of Clinical Oncology, State Key Laboratory of Oncology in South China, Sir YK Pao Center for Cancer and Li Ka Shing Institute of Health Sciences, The Chinese University of Hong Kong, Hong Kong; 3 Department of Chemotherapy, Cancer Center, Qilu Hospital, Shandong University, Jinan, China; 4 Department of Abdominal Surgical Oncology, Cancer Hospital, Peking Union Medical College & Chinese Academy of Medical Sciences, Beijing, China; 5 Microarray Facility, Wellcome Trust Sanger Institute, Cambridge, United Kingdom; National Cancer Center, Japan

## Abstract

**Background:**

*E*sophageal squamous cell carcinoma (ESCC) is highly prevalent in China and other Asian countries, as a major cause of cancer-related mortality. ESCC displays complex chromosomal abnormalities, including multiple structural and numerical aberrations. Chromosomal abnormalities, such as recurrent amplifications and homozygous deletions, directly contribute to tumorigenesis through altering the expression of key oncogenes and tumor suppressor genes.

**Methodology/Principle Findings:**

*T*o understand the role of genetic alterations in ESCC pathogenesis and identify critical amplification/deletion targets, we performed genome-wide 1-Mb array comparative genomic hybridization (aCGH) analysis for 10 commonly used ESCC cell lines. Recurrent chromosomal gains were frequently detected on 3q26-27, 5p15-14, 8p12, 8p22-24, 11q13, 13q21-31, 18p11 and 20q11-13, with frequent losses also found on 8p23-22, 11q22, 14q32 and 18q11-23. Gain of 11q13.3-13.4 was the most frequent alteration in ESCC. Within this region, *CCND1* oncogene was identified with high level of amplification and overexpression in ESCC, while *FGF19* and *SHANK2* was also remarkably over-expressed. Moreover, a high concordance (91.5%) of gene amplification and protein overexpression of *CCND1* was observed in primary ESCC tumors. *CCND1* amplification/overexpression was also significantly correlated with the lymph node metastasis of ESCC.

**Conclusion:**

These findings suggest that genomic gain of 11q13 is the major mechanism contributing to the amplification. Novel oncogenes identified within the 11q13 amplicon including *FGF19* and *SHANK2* may play important roles in ESCC tumorigenesis.

## Introduction

Esophageal cancer is one of the most aggressive malignancies originated in the gastrointestinal tract, and ranks as the sixth leading cause of cancer-related deaths in the world [1]. Its incidence varies greatly among different regions worldwide, with China as a high-risk area. In some districts of north and central China, its incidence exceeds 100 cases/per 100,000 per year [2]. Histologically, esophageal cancer is classified as esophageal adenocarcinoma and esophageal squamous cell carcinoma (ESCC). Most of the cases reported in US are esophageal adenocarcinomas, however in China and other Asian countries, ESCC is the predominant type that accounts for about 90% of all cases. Despite advances in multimodal therapies, ESCC remains a serious cancer-care problem in many countries with very low 5-year survival rates (<30%) [3]. Thus, it is of great clinical value to look for sensitive and specific biomarkers for the early detection and prognosis of this malignancy, as well as novel therapeutic targets.

Genomic amplifications and deletions contribute to human tumorigenesis by altering the expression levels of critical oncogenes and tumor suppressor genes (TSGs). In spite of its high prevalence, ESCC has not been studied as intensively as its adenocarcinoma counterpart. Efforts have been put to identify gross copy number alterations of both ESCC cell lines and tumors, including karyotyping, fluorescence *in situ* hybridization (FISH), conventional comparative genome hybridization (CGH) and loss of heterozygosity (LOH) analyses. According to available data published by now, the most commonly cited chromosomal amplifications in ESCC are 3q, 4q, 5p, 8p, 7q, 9q, 10q21, 11q13-q22, 18p11.3, 20q and 22qtel [4]–[12]. Amplifications harboring oncogenes, e.g. 11q13 (*CCND1*, *EMS1*), 3q26 (*EIF5A2*), 21q22 (*ETS2*), 8q24 *(MYC)*, have been consistently observed in more than one studies [4]–[12]. Chromosomal losses recurrently involve 3p, 5q, 9p, 13q, 18q and 21q, in which target genes such as *FHIT*, *APC*, *RB1* and *CDKN2A* are located [4]–[12]. In recent years, high resolution array-based CGH (aCGH) has been applied to identify target oncogenes and TSGs through defining recurrent gains and losses in various cancers. Until recently, two studies performed aCGH analysis on primary ESCC samples, revealing recurrent, high-level amplifications in 3q27.1, 7p11, 8q21.11, 8q24.21, 11q13.3, 11q22, 12q15–q21.1, 18q11.2, and 19q13.11–q13.12, and homozygous deletions in 4q34.3–q35.1 and 9p21.3 [13]; [14]. However, compared to “pure” ESCC cell lines, primary ESCC samples contain lots of normal cells which may affect aCGH results in different ways. Although several comprehensive whole genome studies on ESCC cell lines has been reported, the cell lines used are mainly originated from Japanese ESCC (TE series) and South African ESCC patients, respectively [15]–[17]. Profiling of multiple ESCC cell lines originated from different high-risk areas in Asian via aCGH will not only allow the identification of recurrent chromosomal changes in Asian ESCC, but also provide valuable insight for future studies using these cells lines as ESCC models.

In this study, we profiled 10 commonly used ESCC cell lines originated from mainland Chinese (EC1, EC18 and EC109), Hong Kong Chinese (HKESC1, HKESC2, HKESC3 and SLMT1) and Japanese (KYSE70, KYSE410 and KYSE520) patients for whole-genome DNA copy number alterations using aCGH analysis. Among identified alterations, amplification of 11q13 is the most frequent gain observed, harboring *FGF19*, *SHANK2* and *CCND1*. We further found that *CCND1* expression was frequently upregulated in primary ESCC tumors, and DNA amplification contributes to its overexpression, which is correlated with lymph node metastasis of primary ESCC tumors.

## Results

### Genomic Profiles of ESCC Cell Lines by 1-Mb aCGH

**Figure 1:**
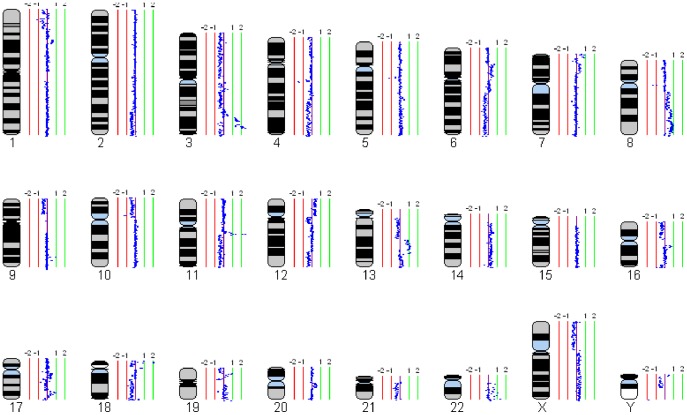
Whole genome profile of an ESCC cell line (EC18) by 1-Mb aCGH. Normalized log2 signal intensity ratios were plotted using SeeGH software. A log2 signal ratio of 0 represents equivalent copy number between the sample and the reference DNA (details in Materials and Methods). Cytoband pattern for each chromosome is shown in the left. Vertical lines denote log2 signal ratios from −2 to +2 with copy number increasing in the right and decreasing in the left. Each dark blue dot represents a single BAC clone.

**Figure 2:**
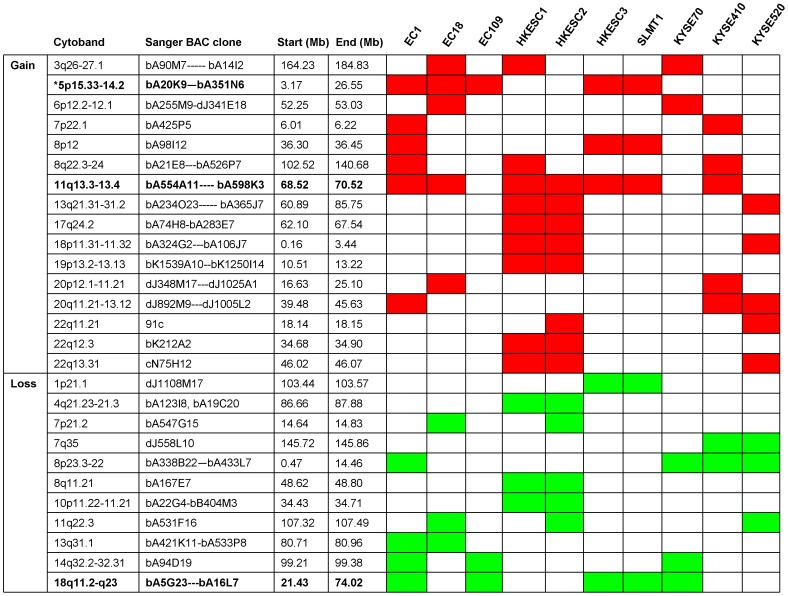
Minimal aberrant regions identified by aCGH. Abnormalities with ≥20% frequency in 10 ESCC cell lines are shown. * Regions with abnormalities in ≥50% cell lines are shown in bold.

Ten ESCC cell lines were analyzed using 1-Mb aCGH (Sanger 3040-BAC/PAC clone array). Signal intensity ratios for each BAC were processed and displayed as log2 plots using SeeGH software [18]. Figure 1 shows the representative SeeGH karyograms of one ESCC cell line (EC18) analyzed, demonstrating the identification of various gains and losses. Other SeeGH karyograms of ESCC cell lines analyzed are shown in Figure S1. Figure 2 summarizes the recurrently altered regions (with log2 ratios more than 1 or less than −1). In general, chromosomal gains were more frequently detected than losses. The most frequent alterations include gain of 11q13 (70%) and complete loss of 18q11-23 (50%). Other gains occurring in three or more cell lines are 3q26-27 (40%), 5p15-14 (50%), 8p12 (30%), 8p22-24 (30%), 13q21-31 (30%), 18p11 (30%) and 20q11-13 (40%). Other losses occurring in three or more cell lines are 8p23-22 (40%), 11q22 (30%) and 14q32 (30%) (Fig. 2).

### Amplification and Overexpression of 11q13 Genes in ESCC Cell Lines

**Figure 3:**
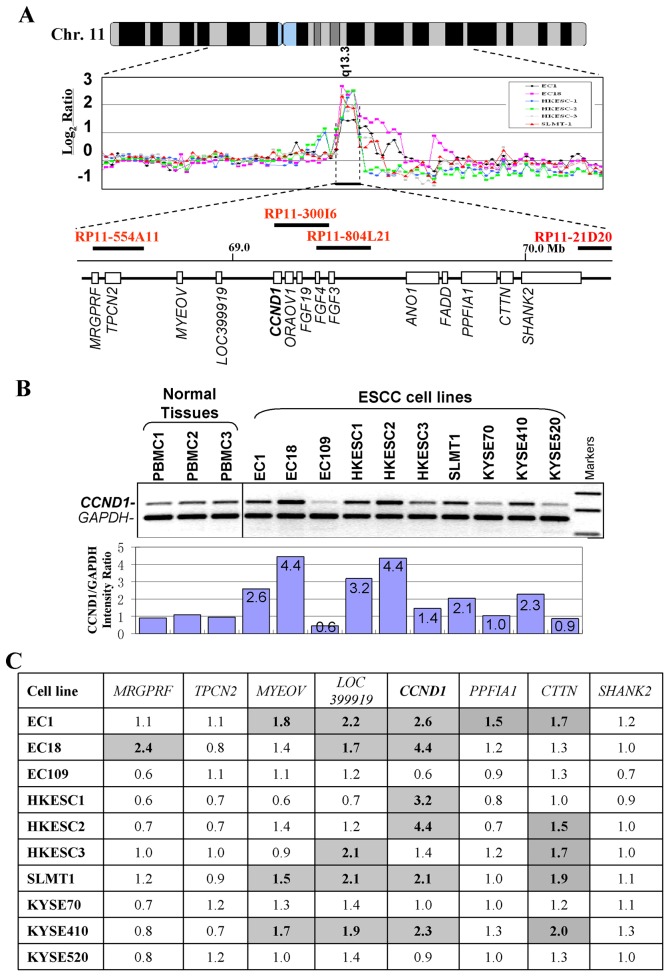
*CCND1* is located at the center of the 11q13 amplicon in ESCC. *A* , aCGH profiles of six ESCC cell lines at the *CCND1* locus. Normalized log2 signal ratios were plotted. Amplifications were defined as log2 signal intensities ≥1. Horizontal lines denote log2 signal ratios from −1 to 3 with copy number increasing upwards. Each black/colorful dot represents a single BAC clone. Names of related BAC clones are also shown. Transcript map of the core 11q13 amplicon is shown in the bottom. ***B***, Semi-quantitative duplex genomic DNA PCR analysis of *CCND1* in 10 ESCC cell lines and 3 normal PBMC samples. Signal intensity ratios of *CCND1*/*GAPDH* are shown. ***C***, Summary of gene copy number changes of several genes within the 11q13 amplicon. Numbers shown in the table are folds of copy numbers of ESCC cell lines relative to the mean values of three PBMC samples. PBMC, peripheral blood mononuclear cell.

Among regions identified with recurrent alterations, amplification of 11q13.3-13.4 is the most frequent gain in ESCC (Fig. 3A), especially in those cell lines derived from Chinese patients (6/7 cell lines, 85%). Several genes with potential oncogenic functions have been identified in this locus, including *CCND1* and *CTTN*. Thus, 11q13 was further investigated for the confirmation of gains and identification of amplified genes, by duplex genomic DNA PCR in 10 ESCC cell lines. *CCND1* is mapped to the center of the 11q13 amplicon, and high-level amplification of *CCND1* was confirmed in 6/10 cell lines (Fig. 3B, C), while *CTTN* exhibited the second highest level of amplification (in 5/10 cell lines).

**Figure 4:**
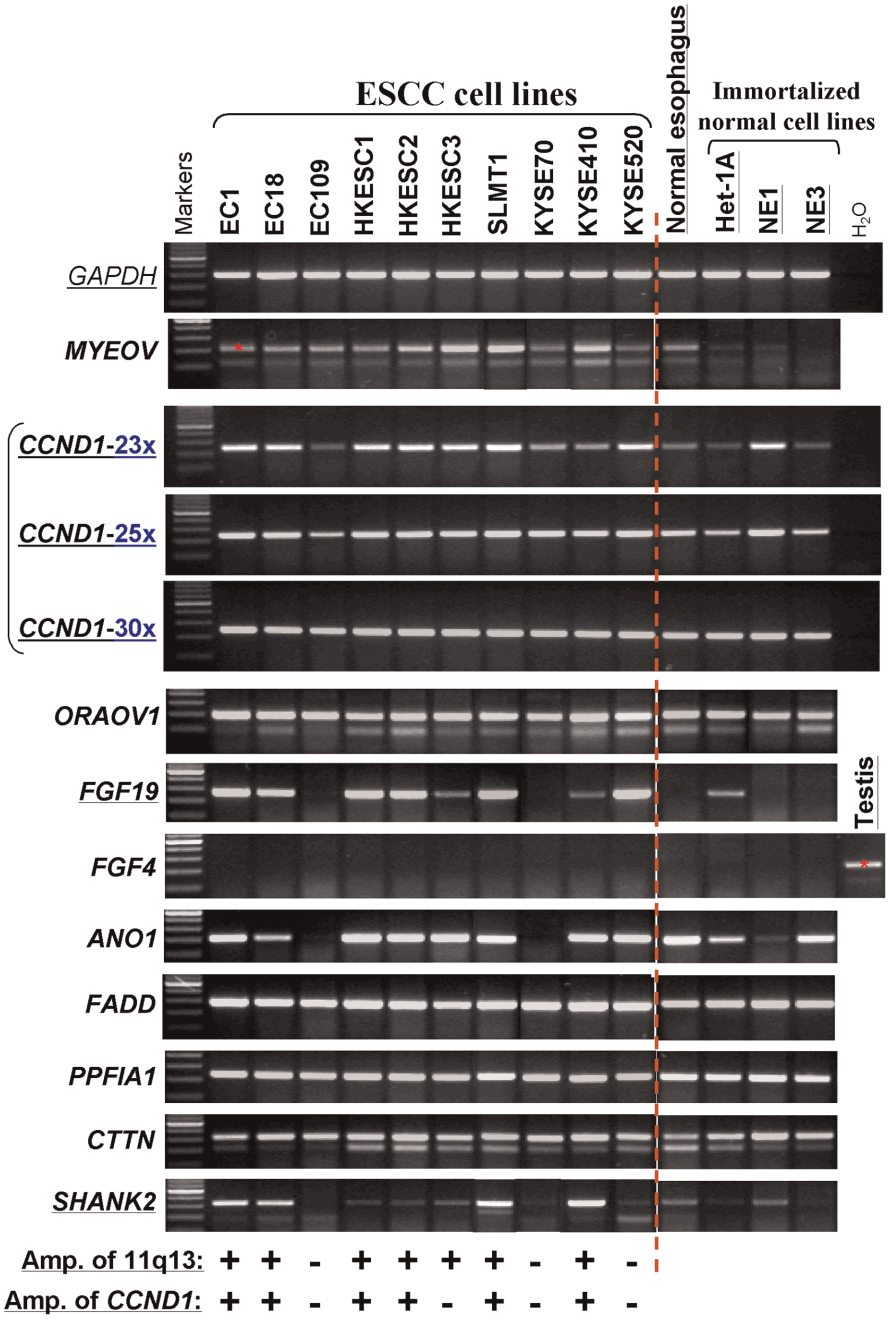
Expression levels of several 11q13 genes around *CCND1* in ESCC cell lines examined by semi-quantitative RT-PCR. The amplification status of 11q13 region by aCGH and *CCND1* by multiplex DNA PCR are listed at the bottom. +, amplified; -, not amplified. 23x, 25x, 30x: RT-PCR cycles. All other genes were examined by RT-PCR with 30 cycles, with *GAPDH* for only 23 cycles.

Several genes around *CCND1* at 11q13 were further examined by semi-quantitative RT-PCR in ESCC cell lines. Results showed that *FGF19* and *SHANK2* were also remarkably overexpressed in ESCC cell lines, while only weakly or not expressed in normal esophagus or immortalized normal cells (Fig. 4), although no obvious amplification of *SHANK2* was detected in these cell lines. *FGF3* (data not shown) and *4* were basically not expressed in any ESCC cell line or normal esophagus. Overexpression of *CCND1* and *CTTN* was also observed in most ESCC cell lines when compared to normal esophagus, although they were generally expressed in normal esophagus and immortalized cells (Fig. 4). Taken together, these data confirmed that 11q13 is the most frequent amplification in ESCC and delineated several genes including *CCND1*, *CTTN*, *FGF19* and *SHANK2,* as potential critical oncogenes affected.

### CCND1 Overexpression in Primary ESCC and its Clinicopathological Association

**Figure 5:**
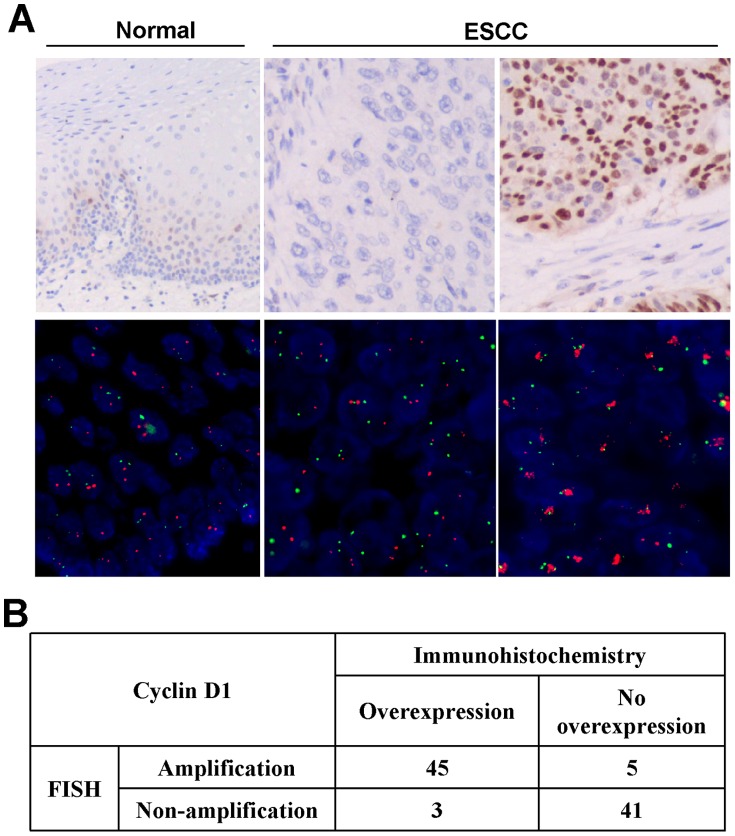
Representative immunohistochemical staining and FISH analysis . ***A***, *Top panels*, immunohistochemical staining for *CCND1*. *Left*, scattered positivity of *CCND1*, especially in the basal layer, is seen in normal esophageal epithelium (original magnification, ×200). *Middle*, a case of ESCC is negative for *CCND1* expression (original magnification, ×200). *Right*, diffuse and strong nuclear staining for *CCND1* in this case of ESCC (original magnification, ×200). *Bottom panels*, FISH analysis. Green signals refer to reference probe of chr 11 centromere while red signals are target probe for *CCND1*. *Left*, unamplified in normal esophageal epithelium, *Middle*, an unamplified ESCC case. *Right*, an amplified ESCC case. ***B***. Results of *CCND1* amplification and expression levels in 94 paraffin-embedded primary ESCC.

Expression of CCND1 protein was further investigated by immunohistochemistry in ESCC tissue microarray (TMA) of 171 primary ESCC and adjacent surgical margin histologic normal esophageal tissues. Cases with >10% of tumor cells showing positive nuclear staining were scored for CCND1 overexpression. Ninety-four of the 171 cases (55%) showed CCND1 overexpression, including 42 cases of grade 1+, 35 cases of grade 2+ and 17 cases of grade 3+ (Fig. 5A). In contrast, only scattered positive cells were found in basal cells of the normal esophageal epithelia.

We then tested the correlation between CCND1 overexpression and clinicopathological features, including tumor size, grade, lymph node metastasis, and patient age. CCND1 overexpression was significantly associated with lymph node metastasis (*p* = 0.006) (Table 1), but not with pT, grade, or patient age (*p*>0.05, respectively). In the cases with CCND1 overexpression, there was no significant difference regarding lymph node metastasis, between CCND1 high-expression groups (grade 2+ and 3+) and the low-expression group (grade 1+) (*p* = 0.37).

**Table 1:** Overexpression of CCND1 was associated with lymph node metastasis.

		*CCND1* expression		p value
		Positive	Negative	
**Differentiation**	Well	18	11	0.62*
	Moderate	48	39	
	Poor	28	27	
**Lymph Node** **Metastasis**	No	35	45	0.006*
	Yes	59	32	

### CCND1 Overexpression Resulting from Gene Amplification but Not Activated β-catenin Signaling

To confirm the gene amplification of *CCND1* in primary ESCC and investigate the association with its overexpression, we applied fluorescence in situ hybridization (FISH) analysis with commercial *CCND1* probe together with CEP 11 probe in 94 cases. *CCND1* was amplified in 50 cases (53.2%) and not-amplified in 44 cases (46.8%) (Fig. 5B). Furthermore, gene amplification by FISH and protein overexpression by immunohistochemistry for *CCND1* showed 91.5% (*κ* = 0.69) concordance.

Immunohistochemically, nuclear staining for β-catenin was positive in only 4/94 cases (4.3%). All β-catenin positive cases were negative for CCND1 by immunohistochemistry.

## Discussion

ESCC is the sixth most fatal cancer worldwide and accounts for 90% cases of all esophageal cancers in China [19]. Although the incidence of ESCC is low in Western countries, this tumor is common in Asia, especially in some regions in China like Henan province and Shantou city [20]; [21]. Like other cancers, environmental and genetic factors contribute to ESCC pathogenesis. Tobacco, alcohol, hot drink/food, dietary deficiency and achalasia have been considered as major environmental risk factors for ESCC, as revealed by epidemiology studies [21]; [22]. Meanwhile, cytogenetic and molecular genetic analyses demonstrated multiple genetic abnormalities in ESCC, including chromosomal losses and gains. Elucidation of these aberrations will lead to better understanding of ESCC pathogenesis, and further develop therapeutics and biomarkers for the prediction of metastasis and prognosis. In this study, we carried out a comprehensive investigation of genomic abnormalities in ESCC by high-resolution array-based CGH, to delineate the minimal chromosomal regions of amplifications and deletions. The results provide detailed pictures of multiple genetic lesions in ESCC genomes, and also give hints for further identification of critical oncogenes and TSGs in this malignancy.

Consistent with previous findings [4]–[14], recurrent gains including 11q13 (*CCND1*, *EMS1*), 3q26 (*EIF5A2*), 21q22 (*ETS2* ) and 8q24 *(MYC)*, and losses including 3p, 5q, 9p, 13q, 18q and 21q with target genes such as *FHIT*, *APC*, *RB1* and *CDKN2A*, were identified in ESCC cell lines in this study. Among amplicons detected, 11q13.3-13.4 is the most frequently amplified chromosomal region. Two genes, *CCND1* and *CTTN* located in this region, were identified with high-level amplifications in multiple ESCC cell lines. While investigated at the mRNA level, we found several genes, including *CCND1 CTTN, FGF19* and *SHANK2*, were frequently overexpressed in ESCC cell lines. Recently, Sawey et al reported that *CCND1* and *FGF19* were two driving oncogenes in hepatocellular carcinoma [23]. In this study, we focused on *CCND1*. Our study confirmed frequent amplification (53.2%) and concordant protein over-expression (51.1%) of *CCND1* in primary ESCC. Moreover, a positive correlation between *CCND1* amplification/overexpression and lymph node metastasis in ESCC was observed, indicating that *CCND1* may serve as a prognostic marker for ESCC, in line with other studies ([24]–[27]. In addition to amplification, CCND1 overexpression may be driven by transcriptional regulation, such as the Wnt signaling pathway. Beta-catenin is a key member of the Wnt signaling pathway, which has been suggested involved in esophageal cancer initiation and progression [28]. In this study, only 4.3% (4 in 94) of ESCC cases were identified with beta-catenin nuclear expression. The results indicate that the genomic gain of 11q13 in ESCC is the primary mechanism resulting in *CCND1* amplification and overexpression. This mechanism has also been reported in other tumor types such as head and neck carcinoma [29], pituitary tumors [30], and breast cancer [31].

CTTN is an actin-associated scaffolding protein, binds and activates actin-related protein complex (Arp2/3) and thus regulates the branched actin networks in the formation of dynamic cortical actin associated structures. *CCND1* and *CTTN* are frequently co-amplified in cancers [32]–[34]. Previously, *CTTN* has been identified as a *bona fide* oncogene located at 11q13 involved in ESCC carcinogenesis, contribute to the metastasis of various caners including ESCC, breast, hepatocellular, and head and neck squamous cell carcinomas [32]–[34]. FGF19, a fibroblast growth factor, together with CCND1, was recently reported to a major driver oncogene in hepatocellular carcinoma [23]. The involvement of FGF19 and other novel oncogenes identified in ESCC pathogenesis and the related molecule mechanisms needs to be further investigated.

Among other frequent chromosomal gains, 3q26-27 is a novel amplicon detected in ESCC. Well-known oncogenes residing in this region include *EVI1*, *EIF5A2* and *PIK3CA*, which have been reported to exhibit potential oncogenic functions. In addition, *TRIO* and *CTNND2* at 5p, *MYC* at 8q22, *KLF5* and *POU4F1* at 13q and *NKX2.2* at 20p were also considered to play oncogenic roles in other tumors [35]; [36] and might contribute to ESCC carcinogenesis. Regions of high-level deletions containing known or candidate TSGs were also detected, including 18q11-23 containing *SMAD2, SMAD4* and *DCC* (EC1, EC109, HKESC3, SLMT1 and KYSE70), 8p22 containing *DLC1* (EC1, KYSE70, 410 and 520) and a region on 14q32 containing *DLK1* and *MEG3* (EC1, EC109 and KYSE70). Other high-level deletions containing candidate TSGs include 4q21.23-21.3 (*MAPK10*, *PTPN13* and *ARGAP24*), 7p21.2 (*DGKB*), 7q35 (*CNTNAP2*), 8q11 (*CEBPD*), 10p11 (*PARD3*), 13q31.1 (*SPRY2*) and 16q22-23 (*ATBF1*). Most of these deleted regions of ESCC have also been reported in one or more other cancer types [37]; [38]. Importantly, genetic/epigenetic disruptions or altered expression levels of some TSG candidates mentioned above, including *SMAD2*, *SMAD4*, *DCC*, *DLC1* and *PARD3,* have been reported in ESCC tumors and cell lines, indicating that aCGH study using multiple tumor cell lines could facilitate the identification of critical cancer genes in human tumors. [39]–[42].

In conclusion, multiple minimal regions of deletions and amplicons were detected in 10 commonly used ESCC cell lines in this study, and several novel oncogenes located in the most frequently amplified region 11q13, including *FGF19*, *SHANK2* and *CCND1*, have been identified. This study provides crucial data for further identification and characterization of critical oncogenes and TSGs involved in ESCC pathogenesis.

## Materials and Methods

### Cell Lines

Ten ESCC cell lines (EC1, EC18, EC109, HKESC1, HKESC2, HKESC3 and SLMT1 are from Chinese patients, while KYSE70, KYSE410 and KYSE520 are from Japanese patients) and three immortalized normal esophageal epithelial cell lines (Het-1A, NE1 and NE3) were used in the study [43]. Cell lines were maintained in RPMI-1640 (Invitrogen, Carlsbad, CA) supplemented with 10% fetal bovine serum (Hyclone, Logan, UT), cultured in 5% CO_2_ at 37°C.

### Tumor Samples

A total of 171 formalin-fixed paraffin-embedded (FFPE) Chinese ESCC samples (from 2007 to 2008) and adjacent surgical margin histological normal esophageal tissues were obtained, after receiving patients’ written informed consents and Institutional Review Board (IRB) approval from the Cancer Hospital, Chinese Academy of Medical Sciences, Beijing, China. All patients were previously untreated (i.e., with no chemotherapy or radiotherapy), with resectable primary tumors. The mean age of patients was 58 years (range 33–78), and male to female ratio was 4.2∶ 1 (138∶ 33). Hematoxylin and eosin (HE)-stained sections were reviewed to confirm the diagnosis and define tumor areas. Tumor stage (pT) and grade were defined according to the current WHO classification of tumors.

### Array CGH Analysis

High-molecular-weight chromosomal DNA was isolated from cell lines using Qiagen kit (Qiagen, Hilgen, Germany). The purity and molecular weight of DNA were examined on agarose gels. 1-Mb resolution whole-genome arrays with 3040 BAC/PAC clones were provided by Sanger Institute, UK (http://www.sanger.ac.uk/Projects/Microarrays/) [44]. Clones details are listed in Ensembl (http://www.ensembl.org/Homo_sapiens/index.html).

Array-CGH was performed with slight modifications [43]–[45]. Briefly, sample DNA (600 ng) was labeled with Cy5-dCTP (Amersham Pharmacia), whereas reference DNA of normal peripheral blood mononuclear cells (PBMCs) from healthy Chinese donors with Cy3-dCTP using the BioPrime Array CGH Genomic Labeling System (Invitrogen, Carlsbad, CA). Unincorporated nucleotides were removed using Purification Module supplied in the labeling system. Labeled samples and normal DNA (50 µl each) were mixed and ethanol precipitated together with 67.5 µl of human Cot-1 DNA (Invitrogen). Then, the mixed DNA were resuspended in 30 µl of hybridization buffer, denatured and prehybridized in a humidity chamber inside a hybridization oven at 5 rpm for 2 hrs at 37°C. The prehybridization mixture was prepared as follows: 80 µl of Herring Sperm DNA (10 mg/ml, Sigma) and 67.5 µl of human Cot-1 DNA (Invitrogen) were precipitated, resuspended in 120 µl of hybridization buffer and denatured for 10 min at 72°C. After prehybridization, the prehybridized probe was added onto the slide, and incubated at 5 rpm for 48 hours at 37°C. Slides were rinsed and washed three times, air-dried and stored at 4°C.

Hybridized slides were scanned using an Axon 4000B scanner (Axon Instruments Inc, Union City, CA) and analyzed with the GenePixPro 4.0 image analysis software where the spots were defined and median fluorescence intensities were calculated. The background subtracted fluorescence intensities were imported into a custom-designed Microsoft Excel spreadsheet template. A particular spot was excluded if the duplicate spots have a difference of >10%. The mean values of duplicate spots were presented in graphical output in the form of mean log2 (Cy5/Cy3) ratio against distance along each individual chromosome (Mb). Chromosome copy number changes were scored as hemizygous loss if the log2 ratio was ranging from −0.2 to −0.7, and homozygous deletion if >−0.7, or genomic gain for log2 ratio of 0.2 to 0.5, and amplification if >0.5. Copy number changes seen on the sex chromosomes were excluded in the analysis.

### Multiplex Genomic PCR

Multiplex PCR permitted a semi-quantitative assessment of DNA amplification/loss. *GAPDH* gene was selected as an internal control. We amplified *CCND1* and the internal control simultaneously. For *CCND1*, a pair of primers (forward: 5′-tgctgcgaagtggaaaccat and reverse: 5′-caacaagttgcagggaagtc) generated a PCR product of 227 bp. For *GAPDH*, a pair of primers (forward: 5′-gcctcactccttttgcagac and reverse: 5′-gatgaccttgcccacagcct) generated a PCR product of 157 bp. PCR reactions were performed in a final volume of 20 µl containing 200 mM deoxynucleotide triphosphates, 2.5 mM MgCl_2_, 50 ng DNA, 0.5 mM each oligonucleotide and 0.5 U AmpliTaq GOLD (Applied Biosystems, Foster City, CA). Reaction conditions were as follows: an initial denature at 95°C for 10 min followed by 30 cycles of 95°C for 30 s, 60°C for 30 s, 72°C for 30 s and a final extension at 72°C for 10 min. PCR products were separated on 2% agarose gels and visualized. All reactions were carried out at least twice in independent experiments.

### Semi-quantitative Reverse Transcription PCR

Total RNA were extracted from cell pellets using TRI Reagent (Molecular Research Centre, Cincinnati, OH). Reverse transcription PCR (RT-PCR) was performed as described [43], using *GAPDH* as a control. The primers for all genes will be provided upon request. The PCR program utilized an initial denaturation at 95°C for 10 min, followed by 30 cycles of reaction (94°C for 30 s, 55°C for 30 s and 72°C for 30 s) (or 23, 25 cycles for CCND1), with a final extension at 72°C for 10 min.

### ESCC Tissue Microarray (TMA)

For each case, both tumor and normal tissues were duplicated with a diameter of 1 mm on a glass slide. Before sample acquisition, HE-stained FFPE slide of each case was observed under a microscope and the locations of typically characteristic morphology of ESCC and surrounding normal tissues were circled. Samples were taken from the circled locations in the paraffin block using the Beecher Instruments Tissue Arrayer (Silver Springs, MD). For each block, two 1 mm cores were punched from the circled regions in the donor block and arrayed on the recipient block to ensure the representation of the samples, and avoid missing information due to a loss of tissue cores. A total of 171 ESCC specimens, 54 specimens of corresponding normal mucosa were arrayed on two recipient blocks. Tissue microarray sections (4 µm) were cut 24 h before immunohistochemistry.

### Automated Immunohistochemistry

Immunohistochemistry was performed with a Ventana Benchmark XT autostainer (Ventana, Tuscon, USA), using monoclonal rabbit anti-human CCND1/cyclin D1 (clone SP4) and monoclonal mouse anti-human β-catenin (Clone β-Catenin-1) from DAKO (Glostrup, Denmark) with Ventana Ultraview kits (Ventana, Tucson, USA). Slides were incubated for 24 min at 37°C with primary antibodies. Diaminobenzidine or 3-amino-9-ethylcarbazole was used as chromogens and slides were counterstained with haematoxylin before mounting. Both chromogens used on regular full sections before TMA testing gave concordant results in terms of both surfaces and intensities of immunostaining. Negative controls were created by omission of primary antibody and replacement with phosphate buffered saline (PBS).

The CCND1 and β-catenin expression levels were determined semi-quantitatively using a four-tiered scoring system, based on the positive nuclear staining fraction of tumor cells (grade 0 = 0–10%; grade 1+ = 11–25%; grade 2+ = 26–50%; grade 3+ = 51–100%). A score of 0 was considered negative while a score of 1+, 2+ or 3+ was considered positive. For β-catenin, staining in cytoplasm may have been present but was not included in the determination of positivity.

### Fluorescence in Situ Hybridization (FISH)

For FISH analysis of *CCND1* gene amplification, two direct-labeled probes were used, Vysis *CCND1*/CEP11 FISH Probe Kit including LSI *CCND1* (11q13) SpectrumOrange against *CCND1* (11q13) and CEP11 SpectrumGreen (Vysis/Abbott, IL, USA) against the centromere of chromosome 11. FISH analysis was done according to “LSI Locus Specific Identifier DNA probes” from Vysis. The *CCND1* gene to chromosome 11 centromere ratio was measured in at least 60 nuclei from tumor cells and an average score was taken. Observing more than two copies of *CCND1* for each chromosome 11 was considered to be a positive sign for *CCND1* gene amplification.

### Statistical Analysis

Statistical analysis was performed using SPSS version 12.0. The association between FISH and IHC results and the clinico-pathological variables are performed using the χ^2^ -test. For all tests, a *p*-value of <0.05 was considered to be statistically significant.

## Supporting Information

Figure S1 **Whole genome profiles of 10 ESCC cell lines by 1-Mb aCGH.** Normalized log2 signal intensity ratios were plotted using SeeGH software. A log2 signal ratio of 0 represents equivalent copy number between the sample and the reference DNA (details in Materials and Methods). Cytoband pattern for each chromosome is to the left for each plot. Vertical lines denote log2 signal ratios from −2 to +2 with copy number increases to the right and decreases to the left. Each black dot represents a single BAC clone.
(TIF)
